# Modulating CD38 enzymatic activity during antibody-based immunotherapy in multiple myeloma: a basic science perspective

**DOI:** 10.3389/fimmu.2026.1769281

**Published:** 2026-04-29

**Authors:** Alberto L. Horenstein, Kristine A. Frerichs, Angelo C. Faini, Niels W. C. J. van de Donk, Fabio Malavasi

**Affiliations:** 1 Laboratory of Immunogenetics, Department of Medical Sciences, Turin, Italy; 2 CeRMS, University of Torino, Turin, Italy; 3 Fondazione Ricerca Molinette, Turin, Italy; 4 Department of Hematology, Amsterdam UMC Location, Vrije Universiteit Amsterdam, Amsterdam, Netherlands; 5 Cancer Center Amsterdam, Cancer Biology and Immunology, Amsterdam, Netherlands; 6 Immunogenetics and Transplant Biology Service, University Hospital “Città della Salute e della Scienza di Torino”, Turin, Italy

**Keywords:** adenosine, CD38, daratumumab, immunosuppression, immunotherapy, inosine, isatuximab, multiple myeloma

## Abstract

**Introduction:**

Multiple myeloma (MM) develops within a profoundly immunosuppressive bone marrow (BM) microenvironment. CD38, a multifunctional ectoenzyme highly expressed on malignant plasma cells, contributes to this niche by degrading nicotinamide adenine dinucleotide (NAD^+^) into ADPR, which fuels adenosine (ADO) production through CD38/CD203a/CD73 enzymatic pathway. CD38_targeting monoclonal antibodies (mAbs), including daratumumab (DARA) and isatuximab (ISA), exert antitumor activity through direct cytotoxicity and immune modulation; however, resistance to these agents remains a major clinical challenge. Understanding how CD38 enzymatic activity and adenosinergic metabolism evolve during therapy is essential for improving treatment efficacy.

**Methods:**

This study investigates (i) the enzymatic functions of CD38, (ii) the *in vitro* effects of DARA and ISA on CD38_mediated NAD^+^ degradation in primary MM cells and a representative MM cell line, and (iii) the *in vivo* dynamics of ADO and its metabolite inosine (INO) in BM and peripheral blood (PB) plasma from MM patients receiving DARA monotherapy.

**Results:**

*In vitro*, both DARA and ISA promoted NAD⁺ degradation with accumulation of ADPR. *In vivo*, ADO concentrations in BM plasma remained consistently in the micromolar range and declined only modestly during treatment, whereas INO progressively increased, leading to a gradual attenuation of the BM -PB gradient. These findings indicate that adenosinergic metabolism remains active during DARA therapy, likely sustained by reduced CD38 expression due to antibody_driven internalization or microvesicle (MV) release, together with adenosine deaminase (ADA) -mediated ADO degradation.

**Discussion:**

Despite CD38_targeted therapy, ADO concentrations in the BM remain well above the activation thresholds of P1 purinergic receptors, suggesting persistent adenosinergic immunosuppression. This sustained ADO production may contribute to a tolerogenic BM niche that promotes immune evasion and therapeutic resistance. These results support the rationale for combining CD38_directed antibodies with agents targeting adenosinergic signaling to enhance antitumor immunity and improve clinical outcomes in MM.

## 1 Introduction

Multiple myeloma (MM) is characterized by the clonal expansion of CD138^+^/CD38^+^ plasma cells within a hypoxic, acidic bone marrow (BM) niche that supports tumor growth through extensive intercellular signaling (1, 2). Despite major therapeutic advances, MM remains incurable (3). A key breakthrough has been the development of monoclonal antibodies (mAbs) targeting surface molecules (4, 5). CD38 rapidly emerged as a promising target due to its high and reproducible expression on MM cells (6). Early CD38-directed approaches showed limited clinical benefit (7–9), whereas the introduction of daratumumab (DARA) and isatuximab (ISA) transformed MM therapy (5, 10). These IgG1κ mAbs bind distinct CD38 epitopes, activate multiple cytotoxic mechanisms, and modulate immunity by enhancing effector responses and depleting immunosuppressive subsets (11–20).

The development of CD38-targeted therapies is complicated by the multifunctionality of the molecule. CD38 is not only a surface marker but also a tightly regulated ectoenzyme expressed in two isoforms (21–24). The predominant type II isoform displays an extracellular catalytic domain that hydrolyzes NAD^+^ to nicotinamide (NAM) and ADP-ribose (ADPR), supporting Ca^2+^ influx, proliferation, and mitochondrial stability (25, 26). Through ADPR generation, CD38 contributes to adenosine (ADO) production and immune suppression (27, 28). The type III isoform, oriented toward the cytoplasm, mainly synthesizes cyclic ADPR (cADPR), regulating intracellular Ca^2+^ signaling (24, 29, 30). CD38 also functions as a co-receptor and adhesion molecule through interactions with CD31 and immune receptor complexes (21, 22, 31–33).

**Figure 1:**
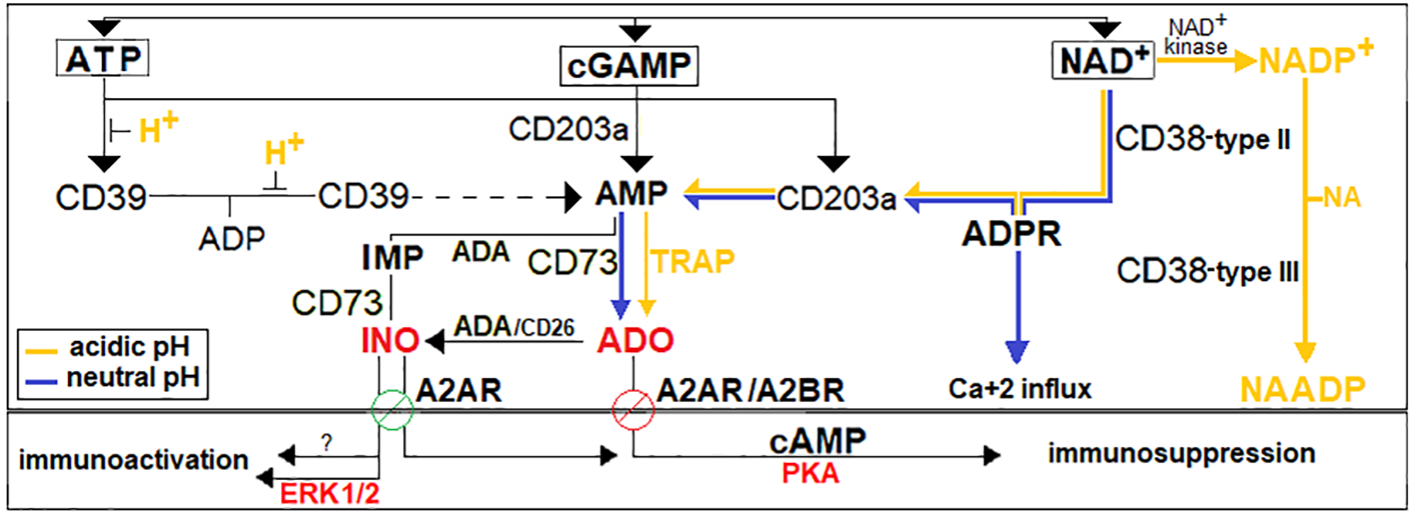
Adenosinergic immune network in the BM niche. Extracellular nucleotides (ATP, NAD^+^, cGAMP) are metabolized by ectonucleotidases including CD38, CD39, CD203a, and CD73 (41). The CD38/CD203a/CD73 axis forms an adenosinergic pathway sequentially converting NAD^+^ to ADPR, AMP, and ADO, respectively, bypassing ATP- and cGAMP-dependent routes (27, 40). The NAD^+^-dependent pathway is highly active in the hypoxic and acidic BM niche, favoring ADO accumulation. ADO activates A2A/A2B receptors, triggering cAMP-mediated immunosuppression. INO, derived from ADO or IMP via ADA, has context-dependent immune effects (40, 42).

CD38 expression persists across all MM stages, likely reflecting its metabolic and immunoregulatory roles (34–37). During disease progression, the BM niche accumulates extracellular nucleotides, including ATP, NAD^+^, and cGAMP, that may promote immune tolerance (38–40) (**Figure 1**). Extracellular NAD^+^—independent of ATP and cGAMP (27, 41)— is sequentially metabolized by CD38, CD203a, and CD73.

These ectoenzymes, expressed by resident cells (21, 27, 37) and by microvesicles (MVs) released from MM cells and surrounding niche elements (6, 43, 44), sustain the CD38/CD203a/CD73 pathway (**Figure 1**), which sequentially generates ADPR, AMP, and ultimately immunosuppressive ADO (27, 37, 45), thereby linking purinergic metabolism to immune evasion (41, 44, 46, 47). In line with previous observations, that AMP formation surpasses ADO clearance (48), we found in a cohort of newly diagnosed MM patients that BM ADO concentrations range from ~25 μM in MGUS/SMM to 25-250 μM in active MM, with particularly elevated levels in ISS stage 3 (45). In this acidic BM environment, ADO activates A2A/A2B P1 receptors, suppressing T and NK cell function and promoting regulatory subsets, thereby fostering a tumor-supportive immunosuppressive environment (16, 49–52).

Based on this background, we hypothesized that the dual role of CD38—as a catalytic signaling receptor and a key component of the adenosinergic network—contributes to immune dysregulation in MM (2, 53–55). We therefore examined how CD38 ligation by therapeutic mAbs modulates ectoenzyme activity. *In vitro* assays using DARA and ISA were performed on primary MM cells (5, 10) and the CD38-rich LP-1 cell line (45, 56), quantifying NAD^+^ consumption and production of cADPR and ADPR, the latter entering the CD203a/CD73 axis to generate ADO, a mechanism we previously demonstrated using co-culture models (27). We then analyzed paired BM and peripheral blood (PB) plasma from MM patients receiving DARA monotherapy (57). Together, these studies demonstrate that CD38-targeting mAbs modulate adenosinergic signaling, supporting the concept that disrupting this pathway may enhance antitumor immunity and improve clinical outcomes in MM.

## 2 Materials and methods

### 2.1 Myeloma cells

The LP-1 (CD38^+^/CD203a^−^/CD73^−^) and BF01 (CD38^+^/CD203a^+^/CD73^−^) myeloma cell lines were established from patients with plasma cell leukemia (6, 56).

Primary MM plasma cells were isolated from BM aspirates by immunomagnetic separation using anti-CD138 monoclonal antibody-coated microbeads (Miltenyi Biotec, Bergisch Gladbach, Germany) (6).

### 2.2 Cells and microvesicle phenotypes

Surface molecule expression on LP-1 and BF01 MM cell lines, primary human BM mononuclear cells, and primary MM cells was analyzed as previously described (6, 45, 57). Cells were resuspended in PBS and examined using a FACSort flow cytometer (Becton Dickinson, San Jose, CA) with CellQuest software; data were processed with FlowJo (TreeStar Inc., Ashland, OR). The phenotype and general characteristics of stabilized cell lines overlapped with those of most MM cells (6). Subsequent experiments employed the LP-1 line (CD138^+^/CD38^+^/CD203a^-^/CD26^+^), maintained in IMDM medium supplemented with 10% FCS (Hyclone), 2 mM L-glutamine and antibiotics.

Primary BM mononuclear cells were isolated by Ficoll-Hypaque density-gradient centrifugation, and analyzed within 24 hours. The frequency of immune cell subsets was assessed by staining 1 x 10^6^ nucleated cells with CD45 (KO), CD56 (PC7), CD138 (APC) (both Beckman Coulter), CD14 (PerCP), CD19 (APC-H7), CD3 (V450) and CD16 (PE) (all BD Biosciences). To assess CD38 expression, we used HuMax-003 (FITC) (Janssen Pharmaceuticals), which binds a CD38 epitope distinct from that recognized by DARA (58). Flow cytometry was performed using a 7-laser BD FACS Celesta (Becton Dickinson) and data were analyzed on a DeNovo software (57). Microvesicles (MVs) were isolated from BM plasma samples of MM patients (n=7) at baseline and during DARA treatment by differential centrifugation, as previously described (59). Briefly, MVs were resuspended in PBS, stained with specific mAbs (20 min at 4 °C), washed with 500 µl PBS, and centrifuged (14,000 *g* for 1 h at 4 °C). The final MV pellet was resuspended in 400 µl staining buffer and analyzed by flow cytometry.

### 2.3 Cell culture conditions

Human MM cells or the LP-1 line were co-cultured *in vitro* with therapeutic CD38 mAbs at 37 °C. Prior to exposure, cells were pelleted (250 g, 5 min, room temperature), resuspended (5 × 10^4^ in 0.5 mL), and incubated (30 min, 37 °C) in AIM V serum-free medium with serial dilutions of DARA, ISA or control human IgG1 mAb (10 μg/mL-100 μg/mL). Cells were then collected for phenotypic analysis, and supernatants were assessed for CD38 enzymatic activity by HPLC, as described above.

### 2.4 Antibodies

Surface molecule expression in myeloma cells was analyzed using monoclonal antibodies (mAbs): anti-CD38 (IB4) (22), anti-CD203a (PC-1, clone 3E8) (60), anti-CD73 (clone CB73), anti-CD26 (clone BT5.9; by A. Bargellesi), and anti-CD138 (clone DL-101). All mAbs were purified in-house as described (61). Conjugated isotype-matched control antibodies were from Beckman Coulter. An affinity purified F(ab’)_2_ rabbit anti-human IgG (H+L) conjugated to Alexa Fluor 488 was obtained from Jackson ImmunoResearch (West Grove, PA, USA).

Daratumumab (DARA), a CD38-targeting IgG1κ mAb approved for MM therapy in 2015 (U.S.) and 2016 (EU), was from Janssen Pharmaceuticals, while isatuximab (ISA) was from Sanofi (11, 62). A human mAb against an innocuous antigen (HIV-1 gp120) was used as an isotype control (22).

### 2.5 Reagents

Nicotinamide adenine dinucleotide (NAD^+^), nicotinamide guanine dinucleotide (NGD^+^), adenosine diphosphate ribose (ADPR), adenosine (ADO), inosine (INO), potassium dihydrogen phosphate (KH_2_PO_4_), and HPLC-grade acetonitrile (ACN) were obtained from Sigma-Aldrich (St. Louis, MO, USA), all analytical grade. Sigma-Aldrich also supplied the inhibitors: kuromanin (CD38 inhibitor); α,β‐Methylene-ADP (APCP, CD73 inhibitor); erythro-9-(2-hydroxy-3-nonyl)adenine (EHNA, ADA inhibitor); levamisole (alkaline phosphatase inhibitor), and dipyridamole (DYP, nucleoside transporter inhibitor).

### 2.6 Patients

#### 2.6.1 Patient selection and study design

Patients were enrolled in a prospective, investigator-initiated, non-randomized, multicenter, open-label study designed to evaluate in part A the safety and efficacy of DARA monotherapy in individuals with relapsed/refractory MM (RRMM) (57). Eligible patients had RRMM after ≥2 prior lines of therapy and presented measurable disease, defined as serum monoclonal protein ≥5 g/L or serum free light chain ≥100 mg/L with an abnormal kappa-to-lambda ratio. Patients were treated intravenously with DARA at a dose of 16 mg/kg, weekly for the first 8 weeks of treatment, then biweekly for the next 16 weeks of treatment, followed by every 4 weeks (57).

PB and BM samples from patients treated with DARA monotherapy in part A of the clinical study were collected at baseline, prior to initiation of DARA monotherapy, and at the time of DARA treatment failure (defined as disease progression [PD] during the first cycle of treatment, less than minimal response after the second treatment cycle, or less than partial response [PR] after the third treatment cycle), or in case of PD after an initial response (PR or better) for evaluation of adenosine (ADO) and inosine (INO) levels. All patients provided written informed consent, and clinical data were collected in accordance with the principles of the Declaration of Helsinki. Ethical approval was granted by the institutional review board of the participating center in The Netherlands.

### 2.7 Chromatographic analyzes by high-performance liquid chromatography

#### 2.7.1 CD38 enzymatic activity assays

##### 2.7.1.1 Substrate conversion assays

LP-1 or BF01 cells were incubated at 37 °C with 100 µM NAD^+^ in 1 mL Hank’s Balanced Salt Solution (HBSS). In selected experiments, LP-1 and BF01 cells were pre-treated with kuromanin (150 µmol/L, 30 min, 37 °C) to inhibit CD38 enzymatic activities.
Extracellular nucleotides were extracted by adding 200 µL acetonitrile (ACN), followed by centrifugation. 500 µL of the supernatant was collected, evaporated to dryness, and analyzed by HPLC. Chromatography was performed using a Beckman Gold 126/166 NM HPLC system equipped with a Synergi 4 µm Polar-RP 80 Å column (150 × 4.6 mm; Phenomenex) and a C18 guard cartridge. The mobile phase consisted of 0.125 M citric acid and 0.025 M KH_2_PO_4_ (pH 5.1) with 8% ACN, at a flow rate of 0.8 mL/min. UV detection was set at 254 nm. HPLC run was done as previously described (45). Individual retention times (Rt, min) for standards were AMP, 2.50; NAD^+^, 3.30; cADPR, 4.00; ADPR, 6.00; and NAM, 7.50 (± 5%). Calibration curves were generated by plotting peak area against known standard concentrations. Quantification was performed by comparing sample peak areas to calibration curves (45, 63). All assays were run in triplicate.

##### 2.7.1.2 Evaluation of antibody effects on CD38 enzymatic activity

LP-1 cells or primary MM cells (5 × 10^4^ in 0.5 mL) were incubated (30 min at 37 °C) in AIM V serum-free medium with serial dilutions of DARA, ISA or control human IgG1 (10 μg/mL-100 μg/mL). Experimental conditions were optimized to maintain reaction linearity and to limit substrate conversion to <25%. Substrates (NAD^+^, NGD^+^, or cADPR; 100 µM) were added and incubated for an additional 15 min at 37 °C on a tilting platform. Reactions were stopped on ice, supernatants (100 µL) precipitated with ACN (1:2, v/v; 4 °C) and filtered using Phree phospholipid removal tubes (Phenomenex). Samples were dried overnight in a vacuum desiccator and reconstituted in 100 µL ultrapure Milli-Q water (Merck Millipore). Ten microliters of each sample were injected into the HPLC system. Peaks were identified using authentic standards, and nucleotide concentrations were determined from calibration curves.

#### 2.7.2 Analysis of adenosine and inosine in bone marrow and peripheral blood samples

This protocol was used to quantify ADO and INO levels in BM and PB samples from MM patients treated with DARA, in order to assess the impact of anti-CD38 mAbs on enzymatic activity. Data are presented as mean ± SD from 3 independent experiments.

##### 2.7.2.1 Sample collection and stabilization

Measurement of ADO requires immediate stabilization to prevent enzymatic degradation (64, 65). BM aspirates (3 mL) were collected (57) and processed as previously described (45). Fresh PB was drawn into ice-cold heparinized Vacutainer tubes containing 0.5 mL STOP solution (45). This solution inhibits adenosine deaminase (ADA), blocks ADO uptake by erythrocytes, and prevents ADO degradation. Samples were centrifuged (700 × g, 5 min, 4 °C), and supernatants were stabilized with ACN (1:3, v/v). Plasma samples were either stored at −80 °C or analyzed immediately by HPLC (45, 66).

##### 2.7.2.2 HPLC analysis

Supernatants were dried using a SpeedVac (Eppendorf) and reconstituted in ultrapure HPLC-grade water (ACILA, Rheinfelden). Standard nucleosides (ADO and INO) were prepared in PBS (pH 7.4), filtered (0.2 µm), and injected (10 µL) into an HPLC system (Beckman Coulter 126/166 NM) equipped with a Synergi Fusion C18 column (4 µm, 150 × 4.6 mm) and C18 guard cartridge (Phenomenex). ADO peak identity was confirmed by ADA peak-shift assay (10 IU/mL), which reduced the signal by 63% ± 15 (n = 10). Concentrations were expressed in µM (45). Chromatographic separations of BM and PB samples used a binary mobile phase consisting of: i) Buffer A: 0.065 mol/L K_2_HPO_4_/KH_2_PO_4_ (pH 6.0), 10 mmol/L tetrabutylammonium dihydrogen phosphate (Sigma), 2% ACN, and ii) Buffer B: 30% ACN in Buffer A. The gradient profile was: 0 min, 0% B; 1 min, 8% B; 10 min, 30% B; 15 min, 2% B; followed by 10 min re-equilibration. Flow rate: 0.8 mL/min; UV detection: 254 nm (63). Retention times (Rt, min): INO, 4.5; ADO, 8.2 (± 5%). Peak areas were quantified using Gold software (Beckman Coulter). The assay showed high linearity for ADO (R^2^ = 0.9972), with LOD and LOQ of 5 ng/mL and 10 ng/mL, respectively (37).

### 2.8 Statistics

Qualitative data were expressed as area percentage (A%). Statistical analyzes were performed using GraphPad Prism version 7.0 (GraphPad Software, San Diego, CA, USA). Comparisons between paired groups were conducted using the Wilcoxon signed-rank test, and results are presented as mean ± standard deviation (SD). A p-value ≤ 0.05 was considered statistically significant.

## 3 Results

**Figure 2:**
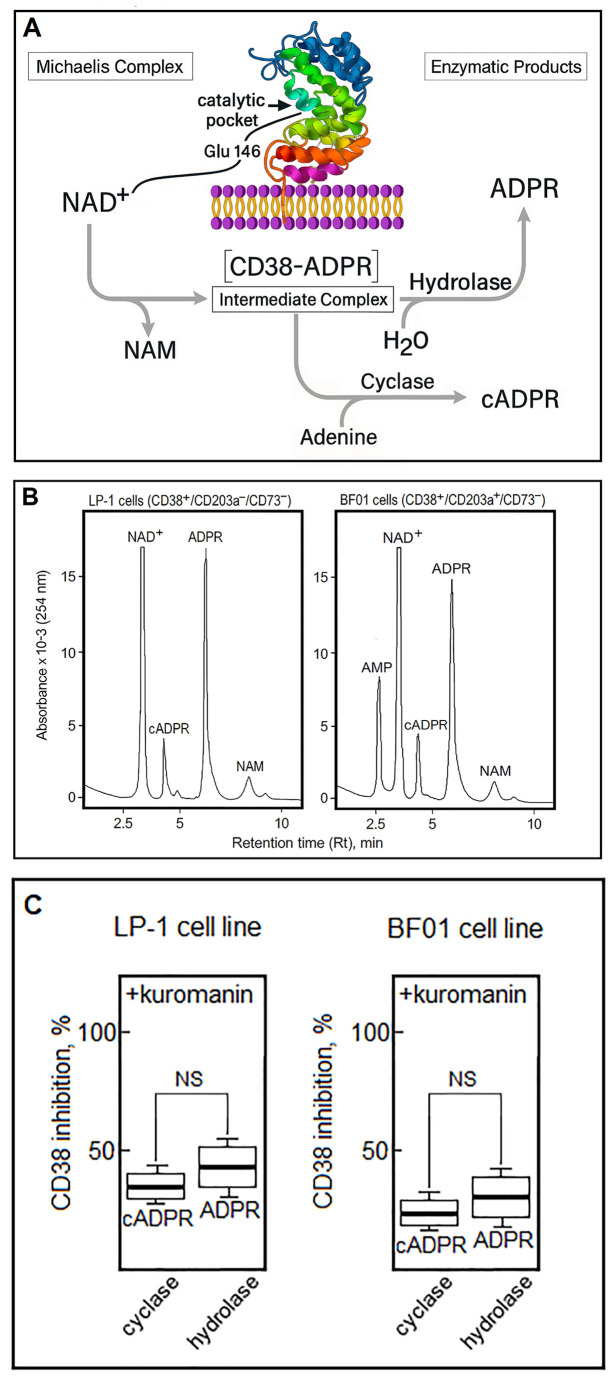
Non-sequential CD38 enzymatic activities in myeloma cell lines. **(A)** Schematic of non-sequential CD38 enzymatic activities within the Glu146-containing catalytic pocket. NAD^+^ binding forms a Michaelis complex, yielding nicotinamide (NAM) and the intermediate [CD38-ADPR] (67, 68). This intermediate can undergo hydrolysis to ADPR or a cyclase reaction with adenine to produce cADPR. Under acidic conditions, base-exchange with nicotinic acid (NA) generates NAADP (69). **(B)** CD38 activity was assessed in LP-1 and BF01 cells incubated with NAD^+^ substrate-excess conditions (100 µM; to ensure linear kinetics and reliable quantification), and product formation (ADPR, cADPR, NAM, AMP) was quantified by HPLC. LP-1 cells produced higher levels of ADPR, consistent with their greater CD38 expression, whereas BF01 cells generated AMP, reflecting CD203a activity. **(C)** Percentage inhibition of CD38 cyclase (cADPR formation) and hydrolase (ADPR formation) activities in LP-1 and BF01 cells in the presence of kuromanin, using NAD^+^ as substrate (n=3 independent experiments).

Evaluation of the *in vitro* effects of anti-CD38 mAbs on the enzymatic functions of the target molecule was conducted using both primary MM cells and a human MM cell line model. The next step was to analyze the *in vivo* consequences of these effects in plasma derived from peripheral blood (PB) and bone marrow (BM) from MM patients treated with DARA. The results were interpreted within the framework of the non-sequential enzymatic mechanism of CD38, which operates through a single catalytic pocket (67–69) (**Figure 2A**).

### 3.1 CD38 enzymatic profiling reveals distinct extracellular NAD^+^-metabolizing activities in MM cell lines

In the LP-1 myeloma cell line (CD38^+^/CD203a^−^/CD73^−^), NAD^+^-glycohydrolase activity predominates and is attributable to CD38 (56), whereas the BF01 line (CD38^+^/CD203a^+^/CD73^−^) displays a broader enzymatic profile (**Figure 2B**). HPLC analysis of NAD^+^ metabolism highlighted distinct activity patterns between LP-1 and BF01 cells. Supernatants from cultured LP-1 cells contained the expected enzymatic products, ADPR and NAM, together with residual unconsumed NAD^+^. ADPR peak areas were quantified by comparison with ADPR standards. The NAD^+^-glycohydrolase activity of LP-1 cells was 3.5 ± 0.04 µmol ADPR per 30 min per 10^6^ cells, with only trace amounts of cADPR. Kuromanin (150 µmol/L, 30 min, 37 °C) affected both CD38 enzymatic activities in LP-1 cells, reducing cyclase activity (NAD^+^ → cADPR) by 25–39% and more strongly suppressing hydrolase activity (NAD^+^ → ADPR) by 45–55% (**Figure 2C**).

These findings confirm that the enzymatic activity detected in LP-1 cells (CD203a^−^) is primarily attributable to native CD38 function, validating their use for subsequent experiments.

### 3.2 DARA and ISA differentially modulate extracellular CD38 enzymatic activities

The effects triggered by CD38 ligation by DARA and ISA mAbs were evaluated on MM plasma cells freshly isolated from BM aspirates of MM patients and, for comparison, with the LP-1 line (CD38^+^/CD203a^−^/CD73^−^). The enzymatic activities were characterized, using: (i) NAD^+^ (to assess both ADPR-Cyclase and cADPR-Hydrolase activities), (ii) nicotinamide guanine dinucleotide (NGD^+^), a non-hydrolyzable NAD^+^ analog (used to measure GDPR-Cyclase activity), and (iii) cADPR (used for direct evaluation of cADPR-Hydrolase activity). Non-consumed substrates and their derived enzymatic products were quantified.

#### 3.2.1 Modulation of NAD^+^-glycohydrolase, ADPR-cyclase, and cADPR-hydrolase activities of CD38 by DARA and ISA in the LP-1 cells

CD38 NAD^+^-glycohydrolase activity (cADPR-Hydrolase and ADPR-Cyclase) was assessed in the LP-1 line following *in vitro* exposure to DARA and ISA. After incubation, CD38 enzymatic activities were evaluated (*see Materials and Methods*) and compared to cells treated with a human, isotype-matched control antibody.

**Figure 3:**
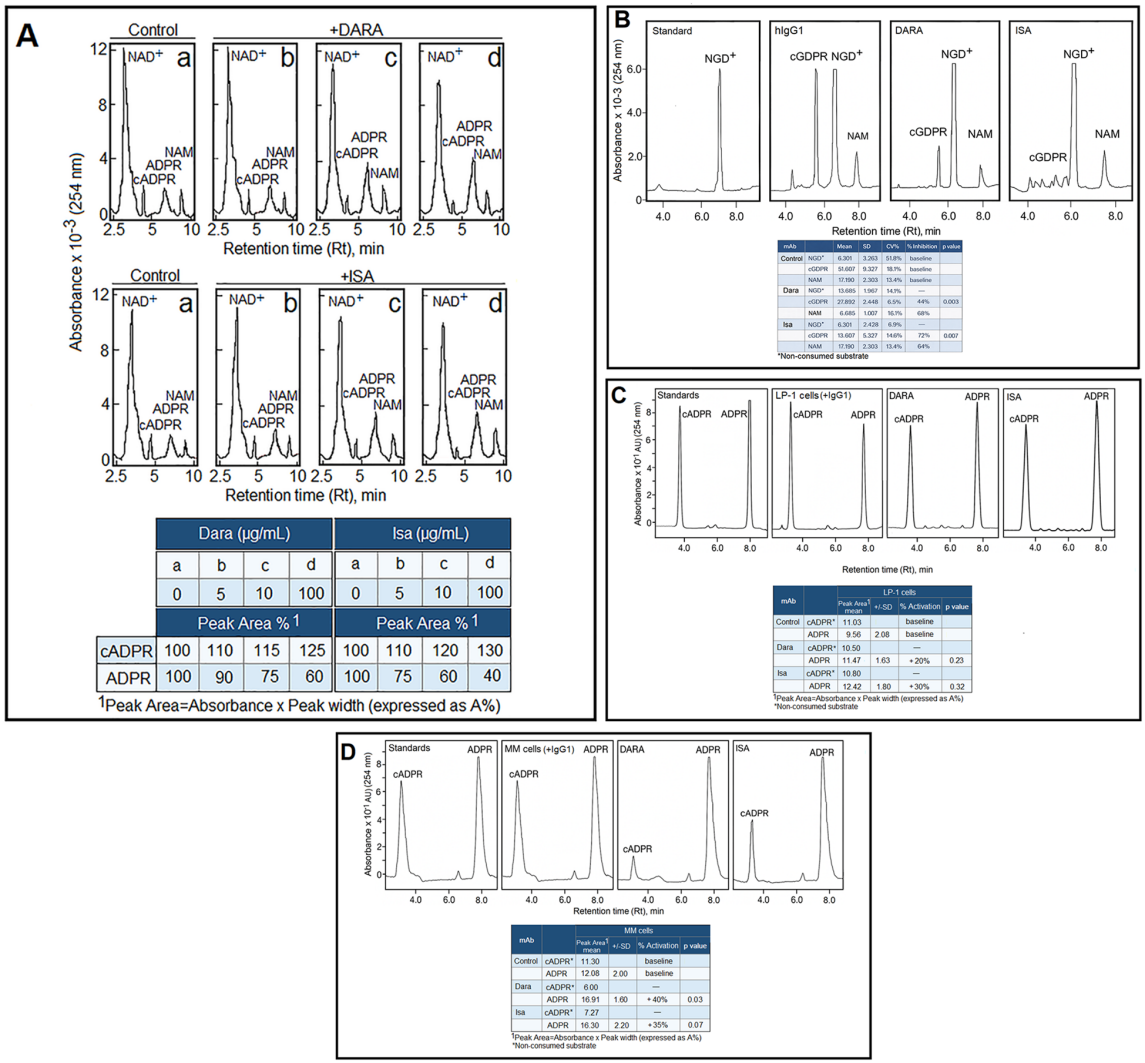
Modulation of CD38 enzymatic activities by DARA and ISA in LP-1 cells and primary myeloma samples. **(A)** Representative HPLC chromatograms of CD38 NAD^+^-glycohydrolase activity in LP-1 cells. **(a)** HPLC analysis of cell supernatants showing residual NAD^+^ and the CD38-derived metabolites cADPR, ADPR, and NAM. **(b–d)** LP-1 cells were pre-incubated with increasing concentrations (5, 10, 100 µg/mL) of DARA, ISA, or control hIgG1 before addition of NAD^+^ (100 µM; substrate-excess conditions to ensure linear kinetics and reliable quantification of product formation). Peak identity was confirmed by co-migration with authentic standards, and quantitative parameters (retention time and peak area) were obtained using Beckman Gold software. The inset table reports the peak-area percentage (A%) of cADPR (cyclase activity) and ADPR (hydrolase activity), in the presence of control hIgG1 or therapeutic anti-CD38 mAbs (DARA and ISA). **(B)** Representative HPLC chromatogram of CD38 NGD^+^-cyclase activity in LP-1 cells. Cells were treated with control hIgG1, DARA, or ISA and then incubated with NGD^+^ (100 µM), a surrogate NAD+ substrate, to quantify cGDPR formation. Peak identity was confirmed by co-migration with authentic standards, and quantitative parameters (retention time, absorbance, peak high, and peak width) were obtained using Beckman Gold software. The inset table reports the percentage inhibition of NGD^+^ cyclase activity in the presence of control hIgG1 or therapeutic anti-CD38 mAbs (DARA and ISA). A two-sample t-test (Welch’s t-test, recommended due to unequal SDs) was applied to assess the significance of the inhibitory activities of DARA and ISA. **(C)** Representative HPLC chromatograms of CD38 cADPR hydrolase activity in the LP-1 line. Cells were treated with control hIgG1, DARA, or ISA and subsequently incubated with cADPR (100 µM; provided by H.C. Lee, University of Singapore) to quantify ADPR formation. Peak identity was verified by co-migration with authentic standards, and quantitative parameters (retention time, absorbance, peak high, and peak width) were obtained using Beckman Gold software. The inset table reports the ADPR peak areas (mean ± SD, n = 3). Even though both antibodies increase ADPR production, the variance and small sample size prevent statistical significance. **(D)** Representative HPLC chromatograms of CD38 cADPR hydrolase activity in primary plasma cells isolated from BM aspirates of MM patients. Cells were treated with control hIgG1, DARA, or ISA and subsequently incubated with cADPR (100 µM; provided by H.C. Lee, University of Singapore) to quantify ADPR formation. Peak identity was verified by co-migration with authentic standards, and quantitative parameters (retention time, absorbance, peak high, and peak width) were obtained using Beckman Gold software. The inset table reports the percentage peak area (A%) corresponding to ADPR (hydrolase activity) in the presence of control hIgG1 or therapeutic anti-CD38 mAbs (DARA and ISA), together with the corresponding statistical significance.

HPLC analysis of LP-1 supernatants showed residual NAD^+^ (Rt = 3.0 min) together with its metabolic products cADPR (Rt = 4.1 min), ADPR (Rt = 5.8 min), and NAM (Rt = 7.1 min), with ADPR representing the major species (**Figures 3A, a**). As expected for CD203a^−^/CD73^−^ cells, no ADO was detected due to the lack of enzymes needed for ADPR-to-ADO conversion. This is fully aligned with our previous work showing in a T-leukemia cell model (27), that ADO in the supernatant of T cells originates from hydrolysis of ADPR to AMP and subsequent CD73-mediated generation of ADO (27, 45).

Changes in ADPR-Cyclase and cADPR-Hydrolase activities were evaluated in the LP-1 line pre-incubated with DARA or ISA (0-100 µg/mL), followed by addition of the NAD^+^ substrate (**Figures 3A, b-d**). The results show that these mAbs differentially modulate the enzymatic functions of extracellular CD38. Specifically, peak levels of cADPR were reduced in the presence of both DARA and ISA, whereas ADPR levels increased following treatment with either antibody. As shown, DARA and ISA (both at 100 µg/mL) inhibited CD38 cyclase activity *in vitro* by ~40% and ~60%, respectively (inset, **Figure 3A**). Additionally, both mAbs enhanced cADPR hydrolysis, resulting in a ~25-30% increase in hydrolase activity. Overall, these findings indicate that CD38 ligation by DARA and ISA promotes NAD^+^ consumption, shifting enzymatic output toward reduced cADPR and elevated ADPR and NAM production, with distinct potencies between the two mAbs.

Modulation of CD38 enzymatic functions by DARA and ISA was confirmed using multiple experimental approaches. Notably, cADPR hydrolysis via the cADPR-Hydrolase function occurs *in vitro* at a rate >100-fold higher than cyclase activity (21). Consequently, the antagonistic effects of CD38 mAbs on cyclase activity were further evaluated using NGD^+^, a non-hydrolyzable NAD^+^ analogue with enhanced GDPR-Cyclase activity. Although not a physiological substrate of CD38, NGD^+^ conversion to cGDPR enables sensitive detection by HPLC owing to product accumulation in the supernatant. HPLC analysis revealed residual NGD^+^ (Rt = 6.8 min), together with cGDPR (Rt = 5.7 min) and NAM (Rt = 7.7 min) (**Figure 3B**). No additional peaks were detected, indicating that anti-CD38 mAbs do not induce alternative reaction pathways or generate novel metabolites. Analysis of CD38 catalytic outputs revealed that both antibodies reduced the formation of cGDPR product, with DARA producing moderate inhibition (−44%; p=0.003) and ISA exerting a more pronounced suppression (−76%; p=0.007), as shown in the inset of **Figure 3B**. Coefficients of variation were low for most conditions, indicating high reproducibility.

Using cADPR as a substrate, we assessed CD38 cADPR-Hydrolase activity in LP-1 cells (**Figure 3C**). Both antibodies induce cADPR consumption to increase ADPR production, consistent with a shift toward CD38 hydrolase activity and a 2–3‑fold rise in the ADPR/cADPR ratio. **Figure 3C** (inset) shows that ADPR production in LP-1 cells increases consistently following anti-CD38 antibody treatment: mean ADPR peak areas rose from 9.56 ± 2.00 in the IgG1 control to 11.47 ± 1.60 with DARA and 12.42 ± 1.80 with ISA. Neither antibody reached statistical significance compared with control (DARA: p = 0.23; ISA: p = 0.32), although ISA displayed a clearer upward trend consistent with enhanced CD38 hydrolase activity.

Together, these findings demonstrate: (i) modulatory effects of CD38 mAbs on ADPR-Cyclase and cADPR-Hydrolase activities in the LP-1 line, and (ii) antibody-specific differences in regulating CD38 catalytic functions.

#### 3.2.2 DARA and ISA increase cADPR-hydrolase activity of CD38 in primary MM cells

The results obtained in LP-1 cells were confirmed in purified primary plasma cells (CD138^+^/CD38^+^/CD203a^+^/CD73^−^/CD26^+^) (37, 45) isolated from MM patients (**Figure 3D**). These MM cells efficiently converted cADPR into ADPR and NAM, while the absent or low CD73 expression characteristic of malignant plasma cells accounted for the lack of detectable ADO formation.

Both DARA and ISA enhanced cADPR-Hydrolase activity, increasing mean ADPR peak areas from the IgG1 control value (12.08 ± 2.00) to comparable levels (16.91 ± 1.60 and 16.30 ± 2.20, respectively) (inset, **Figure 3D**). Consistently, both antibodies elevated ADPR production in MM cells, with DARA reaching statistical significance (p = 0.03) and ISA showing a near-significant trend (p = 0.07). The magnitude and reproducibility of these changes align with the expected biochemical consequences of CD38 engagement by DARA and ISA and remain biologically meaningful within the context of purinergic remodeling.

The conversion of (i) NAD^+^ to ADPR, (ii) NGD^+^ to cGDPR, and (iii) cADPR to ADPR in the presence of DARA and ISA led to the following conclusions from *in vitro* experiments: 1) With NAD^+^ as substrate, CD38 generated ADPR, cADPR, and NAM. Both DARA and ISA enhanced hydrolase activity, increasing NAD^+^ conversion to ADPR. 2) With cADPR as substrate, both antibodies increased cADPR-hydrolase activity, as expected for agonistic antibodies. Moreover, both inhibited CD38 cyclase activity. 3) These inhibitory effects on cyclase function were corroborated using NGD^+^ as a CD38 substrate.

### 3.3 DARA treatment lowers ADO and elevates INO to concentrations within the receptor-activating range in MM patients

To determine whether the *in vitro* findings translate to the clinical setting, we next quantified ADO and INO levels in paired BM and PB samples collected from 7 MM patients treated with DARA monotherapy (**Table 1**). Among these patients, 3 experienced disease progression (PD) during the first treatment cycle, 2 had stable disease, and 2 developed PD after having achieved response (1 partial response [PR] and 1 very good partial response [VGPR]) (57). Patient samples were obtained at baseline (PRE-START phase) and at the time of DARA treatment failure (PD or insufficient response during DARA treatment). As expected, DARA treatment reduced CD38 expression on BM-localized MM cells as well as on monocytes, B cells, T cells and NK cells, compared with baseline (**Supplementary Figure S1**).

**Table 1:** Baseline characteristics of MM patients.

Patient characteristic	Value
Age, median (range)	71 years (62-80)
Sex, n (%)	Female: 2 (29%) Male: 5 (71%)
WHO Performance Score, n (%)	0: 1 (14%) 1–2:6 (86%)
Extramedullary Plasmacytomas, n (%)	No: 6 (86%) Yes: 1 (14%)
Monoclonal Heavy Chain, n (%)	IgG: 6 (86%) Light chain only: 1(14%)
Light Chain Type, n (%)	Kappa: 5 (71%) Lambda: 2 (29%)
Time Since First Treatment, median (range)	6.5 years (2.8-10.9)
Prior Lines of Treatment, median (range)	4 (3-11)
ISS at Registration, n (%)	Stage II: 2 (29%) Stage III: 3 (43%) Unknown: 2 (29%)
Cytogenetic Risk Profile, n (%)	High risk: 2 (29%) Standard risk: 1 (14%) Not available: 4 (57%)

Biochemical Parameters	Median (Range)
Absolute Neutrophil Count (×10^9^/L)	2.41 (1-4.14)
Hemoglobin (mmol/L)	7.1 (5.4-8.2)
Platelet Count (×10^9^/L)	200 (69-360)
Creatinine (μmol/L)	85 (73-97)
Calcium (mmol/L)	2.35 (2.28-2.44)
LDH (U/L)	170 (135-231)
Plasma Cells in Bone Marrow (%)	20 (5-75)

Demographic, clinical, and biochemical parameters recorded prior to treatment.

BM and PB samples were processed for HPLC analysis. Because of the short *in vivo* half-life of ADO, samples were treated at collection with a blocking solution to prevent ADO deamination to INO and cellular uptake. This procedure ensured complete recovery of standard nucleosides from ACN-treated samples (45).

ADO and INO levels in BM and PB samples from all MM patients, collected at baseline and at the time of DARA treatment failure, are reported in **Table 2**. As expected, BM ADO concentrations showed substantial inter-patient variability, consistent with previous reports describing wide fluctuations among MM patients (45). Earlier work demonstrated that BM cells from MM aspirates express CD38, CD203a, CD73, and CD26, with malignant plasma cells characterized by high CD38 expression and variable CD203a positivity, whereas CD73 is largely restricted to stromal and osteogenic cells (45).

**Table 2:** Individual patient values for ADO and INO concentrations in bone marrow and peripheral blood at baseline (pre-start) and at DARA treatment failure.

Patient	Treatment	BM ADO (µM)	BM INO (µM)	PB ADO (µM)	PB INO (µM)	BM ADO/ (ADO+INO)	BM INO/ (ADO+INO)	Best Response1
034	PRE-START	38.70	26.70	20.59	11.38	0.592	0.408	VGPR
	DARA	21.12	35.40	13.37	12.60	0.374	0.626	
042	PRE-START	36.66	53.10	10.82	24.73	0.408	0.591	PD
	DARA	25.75	37.30	6.70	15.56	0.409	0.592	
044	PRE-START	19.86	26.72	23.00	17.86	0.426	0.573	SD
	DARA	16.56	30.82	17.63	18.17	0.350	0.651	
051	PRE-START	35.76	7.84	20.02	12.18	0.820	0.180	PR
	DARA	26.48	16.84	7.81	4.99	0.611	0.389	
055	PRE-START	18.65	12.52	19.59	6.43	0.598	0.402	SD
	DARA	21.59	26.70	19.81	24.82	0.447	0.553	
065	PRE-START	19.76	19.79	20.18	26.86	0.501	0.492	PD
	DARA	23.85	23.09	25.08	30.41	0.508	0.499	
066	PRE-START	18.97	26.81	14.62	9.08	0.414	0.586	PD
	DARA	15.85	25.73	12.55	7.92	0.381	0.619	

Paired BM and PB concentrations of ADO and INO at baseline and at DARA treatment failure are shown for each patient, together with derived ADO/(ADO + INO) and INO/(ADO + INO) ratios and best response to DARA therapy (IMWG criteria).

1***Patient samples were collected either at baseline or during non-response to DARA. Samples 034 and 044 were obtained at progression after 8 cycles; samples 042, 065, and 066 at early progression after the 1st cycle; and samples 051 and 055 at non-response after 2 cycles. Due to the trial design, SD after 3 cycles (<PR) was considered treatment failure (PD), precluding assessment of PFS for DARA monotherapy (57). Abbreviations: VGPR, very good partial response; PR, partial response; SD, stable disease; PD, progressive disease; IMWG, International Myeloma Working Group; PFS, progression-free survival.

In line with this profile, analysis of primary MM cells isolated from BM aspirates (**Supplementary Figure S2A**) confirmed predominant CD38 expression, variable CD203a levels, and absence of CD73. Re-evaluation of a previously analyzed BM aspirate supernatant from a patient with exceptionally high ADO levels (45), provides additional context for the upper range of purinergic activity observed in MM. Using the analytical framework applied in the present study, this outlier sample was confirmed to contain ADO, INO, and intermediate metabolites such as ADPR and AMP (**Supplementary Figure S2B**).

Within the current cohort (**Table 2**), individual patient patterns further underscored this variability: patients 055 and 065 showed modest ADO changes within assay variability, accompanied by proportional INO increases consistent with ADO→INO conversion; patient 042 exhibited decreases in both metabolites, although the unusually high baseline INO level suggests pre-existing metabolic heterogeneity; and in patient 066, ADO decreased while INO remained essentially unchanged, a pattern compatible with the very low INO concentrations detected in both BM and PB, potentially reflecting reduced CD26/ADA activity.

**Figure 4:**
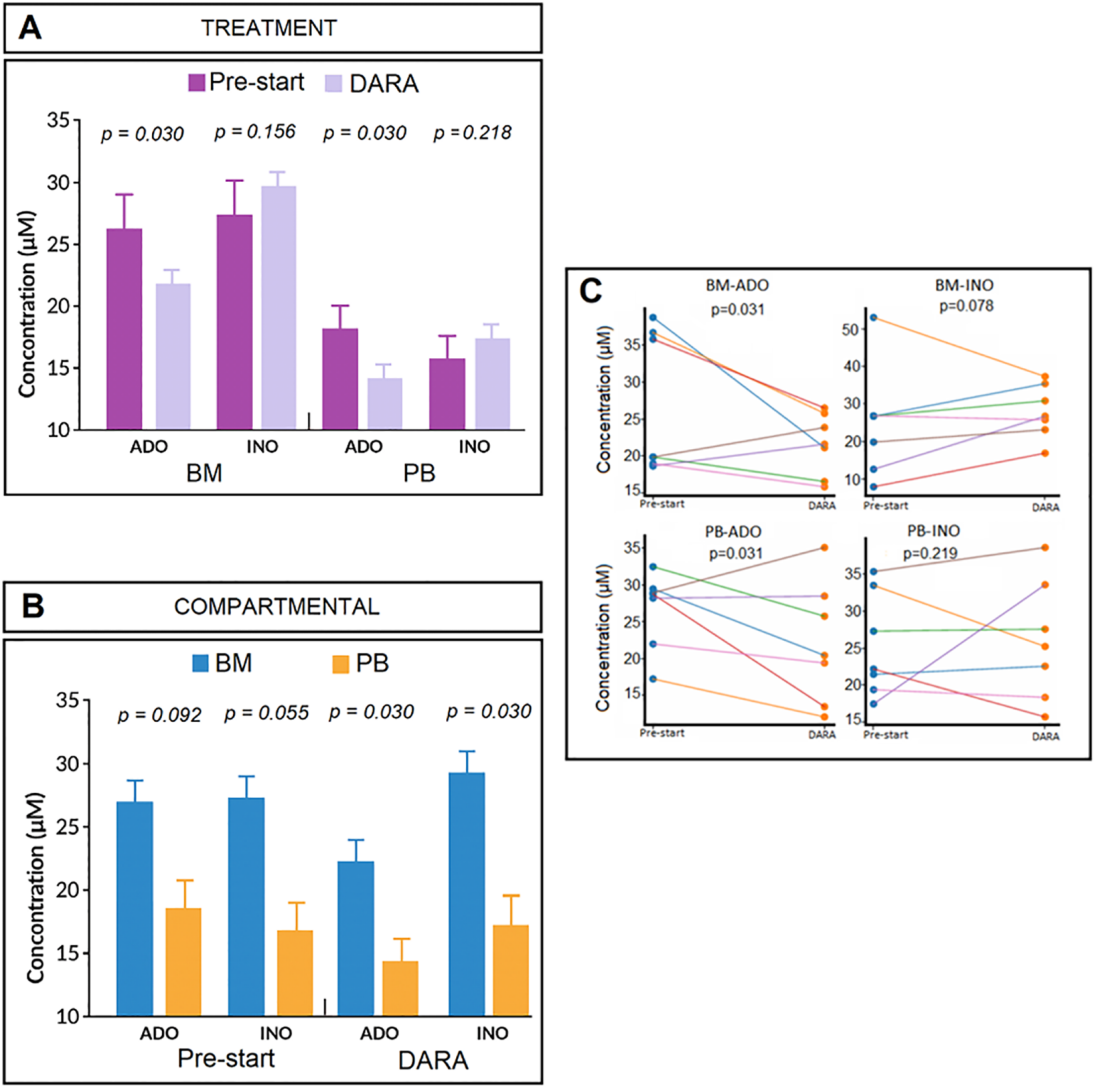
Purinergic metabolite modulation before and during DARA therapy. ADO and INO concentrations (µM) were quantified in paired BM and PB plasma from MM patients (n=7) collected before treatment initiation (PRE-START) and at DARA treatment failure (DARA→PD) (57). **(A)** shows comparisons between treatment phases (PRE-START vs. DARA→PD), and **(B)** shows comparisons between compartments (BM vs. PB). Data are presented as mean ± SD. **(C)** shows that BM-ADO levels decreased significantly during treatment (p = 0.031), BM-INO displayed a non-significant trend toward increase (p = 0.078), PB-ADO levels also declined significantly (p = 0.031), and PB-INO concentrations showed no significant change (p = 0.219). Each line represents an individual patient; Wilcoxon signed-rank test, p ≤ 0.05 considered significant.

Across the cohort, BM plasma analysis (**Table 2**) revealed marked variability in ADO concentrations between treatment phases: PRE-START (mean ± SD: 26.36 ± 8.77 μM; range: 18.65–38.70 μM) and DARA treatment failure (mean ± SD: 21.91 ± 4.07 μM; range: 15.85-26.65 μM) (**Figure 4A**). This represents a statistically significant reduction in BM ADO levels at the time of DARA treatment failure (p = 0.030) (**Figure 4A**).

Nevertheless, BM ADO levels remained within the micromolar range, exceeding P1-receptor activation thresholds (49). This profile is likely sustained by reduced CD38 activity—potentially due to vesiculation, internalization, or trogocytosis— while INO progressively increases, leading to a gradual attenuation of the BM–PB gradient. The pattern indicates an active ADO-to-INO conversion in the presence of DARA. Although ADA activity was not directly measured, CD26—the membrane anchor for ADA—was detected on patient-derived MVs (**Supplementary Figure S3**), suggesting that vesicular compartments may contribute to this metabolic shift. INO concentrations in BM plasma showed modest increases across treatment phases: PRE-START (mean ± SD: 26.79 ± 14.91 μM; range: 18.64-53.10 μM) and DARA treatment failure (mean ± SD: 28.85 ± 7.31 μM; range: 15.25-25.10 μM), although this difference did not reach statistical significance (p = 0.156) (**Figure 4A**).

Similarly, PB samples revealed distinct treatment-related trends: ADO concentrations significantly decreased from PRE-START (mean ± SD: 18.41 ± 4.26 μM; range: 10.82-23.00 μM) to DARA treatment failure (mean ± SD: 14.19 ± 6.16 μM; range: 6.70-25.12 μM) (p = 0.030), while INO concentrations increased slightly from PRE-START (mean ± SD: 15.43 ± 7.12 μM; range: 6.93-26.80 μM) to DARA treatment failure (mean ± SD: 16.49 ± 8.73 μM; range: 4.99-30.41 μM) (p = 0.218) (**Figure 4A**).

To assess compartmental differences (BM vs PB) in ADO and INO distributions, we compared ADO and INO levels in paired BM and PB samples across treatment phases (PRE-START and DARA treatment failure) (**Figure 4B**). Specifically, baseline ADO levels showed a non-significant downward trend from BM to PB (mean ± SD: 26.36 ± 8.77 µM vs 18.41 ± 4.26 µM; p = 0.092). Likewise, INO levels at baseline decreased non-significantly from BM to PB (mean ± SD: 26.79 ± 14.91 µM vs 15.43 ± 7.12 µM; p = 0.055). At the time of DARA treatment failure, both ADO and INO concentrations were significantly higher in BM compared to PB: ADO (mean ± SD: 21.91 ± 4.07 µM vs 14.19 ± 6.16 µM; p = 0.030) and INO (mean ± SD: 28.85 ± 7.31 µM vs 16.49 ± 8.73 µM; p = 0.030) (**Figure 4B**).

As shown in **Figure 4C**, paired BM and PB measurements obtained before and after DARA treatment revealed a significant reduction in ADO levels in both compartments (BM ADO: p = 0.031; PB ADO: p = 0.031). This decline indicates reduced ADO accumulation following CD38 blockade, likely reflecting decreased CD38 surface expression. In contrast, INO levels showed heterogeneous, non-significant changes (BM INO: p = 0.078; PB INO: p = 0.219), aligned with variable ADA activity and the complex cellular composition of the BM niche. Analysis of individual patient profiles (**Figure 4C**) suggests that DARA may transiently increase ADPR-derived ADO, which subsequently declines but remains within the micromolar receptor-activating range at treatment failure, whereas INO continues to accumulate.

BM samples at time of treatment failure displayed lower ADO/(ADO + INO) and higher INO/(ADO + INO) ratios (**Table 2**). These normalized ratios better reflect substrate–product balance and are more informative than absolute nucleotide levels, which are affected by inter-patient and sampling variability. Compared with PRE-START samples (ADO fraction, mean ± SD: 0.537 ± 0.148), BM samples obtained at time of treatment failure to DARA monotherapy showed a significantly reduced ADO contribution (0.440 ± 0.092; p = 0.047), accompanied by a reciprocal increase in the INO fraction (0.463 ± 0.145 vs. 0.560 ± 0.091; p = 0.0469).

**Figure 5:**
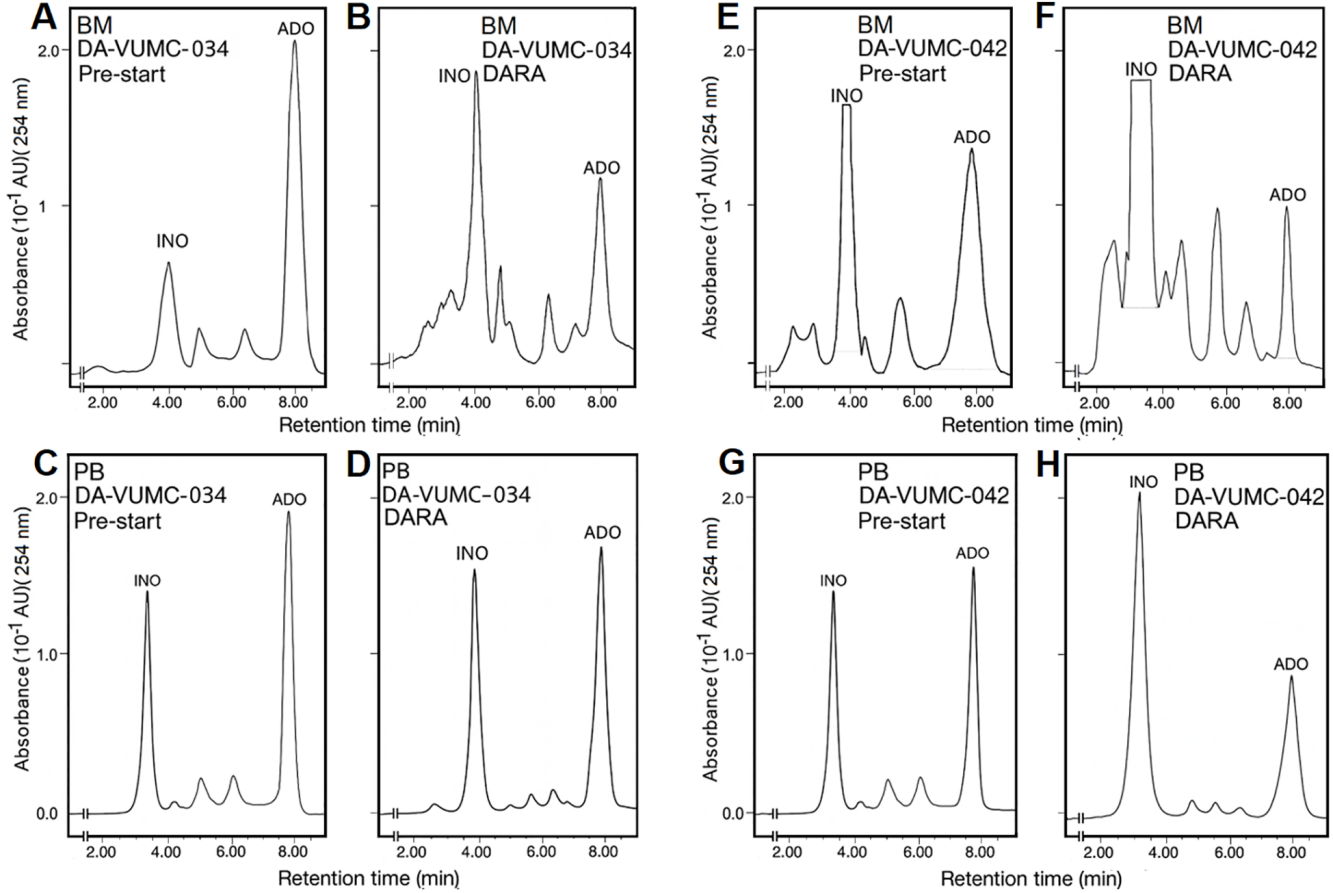
Chromatographic analysis of ADO and INO in BM and PB samples from two representative subjects. **(A–D)** show BM and PB chromatographic profiles of ADO and INO from subject 034 before treatment initiation (Pre-start) and at disease progression (PD) after 8 DARA treatment cycles. **(E–H)** display the corresponding profiles from subject 042, who experienced disease progression after 1 treatment cycle (57). BM and PB samples were processed for HPLC and retention times (Rt) together with peak intensities illustrate differential purine metabolism at baseline and at the time of DARA treatment failure.

Representative HPLC profiles from 2 of the 7 DARA-treated MM patients are shown in **Figure 5**. Data from patient 034 (**Figures 5A–D**; VGPR) and patient 042 (**Figures 5E–H**; primary refractory to DARA) demonstrated reliable detection of ADO and INO in both BM and PB. Patterns of disease evolution varied markedly across the cohort. Patient 042 showed disease progression after only one 28-day treatment cycle, consistent with primary resistance, whereas other patients experienced later progression, including PD after eight treatment cycles (~7–8 months of continuous DARA exposure). These divergent clinical courses underscore the heterogeneity of treatment responses and the coexistence of primary and acquired resistance mechanisms within the cohort (57). In line with the overall cohort, the BM microenvironment displayed higher baseline ADO and INO than PB, reflecting localized accumulation and enhanced purinergic signaling. At the time of DARA failure, the BM-to-PB ADO gradient persisted but was attenuated, although still above immunoregulatory thresholds [e.g., exceeding the Km of ADO receptors (37, 49)], whereas INO remained elevated in BM and became more variable in PB.

Interpretation of adenosinergic activity in MM must consider that the CD38/CD203a/CD73/CD26 ectoenzymatic pathway is discontinuously distributed among malignant plasma cells, stromal and bone-derived cells, and immune elements within the BM niche (37, 45). Consequently, phenotypic changes in any single population cannot be directly extrapolated to the overall ADO output.

In this context, DARA engagement induces membrane remodeling and promotes the release of MVs enriched in CD38 and co-clustered adenosinergic ectoenzymes (CD203a, CD73, and CD26), as previously described (6, 44, 59). To capture the integrated adenosinergic phenotype underlying the purinergic remodeling, we analyzed MVs isolated from BM samples collected before treatment and during DARA therapy (**Supplementary Figure S3**). Flow-cytometric profiling showed that several ectoenzymes involved in ADO production and its conversion to INO were enriched on MVs. Significant differences emerged between baseline and on-treatment samples, consistent with the proposed model of CD38 reorganization under therapeutic pressure.

Together, these findings support a role for MV-associated ectoenzymes in shaping ADO levels during prolonged DARA therapy. The concomitant rise in INO further indicates active ADO catabolism *in vivo*. Combined with the DARA-induced increase in ADPR observed *in vitro* (**Figure 3D**), a precursor in the ADO-generating pathway (27), the data converge on a model in which (i) CD38 contributes to the conversion of NAD^+^ into immunosuppressive ADO within the hypoxic BM niche of MM (27, 45), and ii) modulation of CD38 activity by DARA may translate into altered purinergic metabolism *in vivo* (70). Overall, enzymatic, cellular, and patient-derived data reveal a dynamic purinergic landscape during DARA exposure, characterized by sustained ADO production, increased conversion to INO, and MV-mediated enzymatic support, collectively indicating a metabolically adaptable and immunosuppressive BM microenvironment at the time of treatment failure.

## 4 Discussion

Extracellular CD38 is a multifunctional enzyme integral to NAD^+^ metabolism that predominantly generates the immunomodulatory metabolite ADPR (21). In MM, mAbs targeting CD38 have become a cornerstone of immunotherapy. Here, we investigate how these mAbs modulate CD38 enzymatic activity *in vitro* and influence therapeutic outcomes. Translating *in vitro* enzymatic profiles to *in vivo* settings is challenging due to the complex interplay between CD38-driven NAD^+^ metabolism and adenosinergic signaling. To address this, we integrated *in vitro* analyzes from a MM cell line and primary MM cells with *in vivo* data from BM and PB plasma collected at baseline and at DARA treatment failure. This integrative approach reveals that mAb-mediated modulation of CD38 activity can alter adenosinergic metabolite profiles, potentially influencing therapeutic response and resistance.

The therapeutic mAbs DARA and ISA act as surrogate ligands, binding CD38 at epitopes distinct from the NAD^+^-binding pocket and inducing allosteric conformational changes (54, 71). *In vitro*, both mAbs markedly suppress ADPR-Cyclase activity (residual activity: ~1.4% for DARA; ~0.8% for ISA), possibly by hindering adenine access, while enhancing cADPR-Hydrolase activity via facilitated water entry (**Figure 2A**), in line with an agonistic modulation (2). ISA exerts stronger cyclase inhibition than DARA, likely due to epitope-specific binding differences (54). The antibodies also differ in CD38 trafficking: ISA promotes internalization without shedding (72, 73), whereas DARA induces polarized clustering and release of adenosinergic MVs (6, 44). These findings highlight the epitope- and context-dependent nature of CD38 modulation by therapeutic antibodies.

Beyond its primary role in converting NAD^+^ to ADPR, extracellular CD38 generates only minimal cADPR output, which plays a limited role in Ca^2+^ signaling in MM. Notably, cADPR, intracellularly produced by the potent cyclase SARM1 (74), is largely unaffected by surface CD38 activity. Because DARA and ISA act exclusively at the cell membrane, their impact on cADPR-dependent signaling is likely negligible. Therefore, the notion that CD38-targeting mAbs alleviate immunosuppression by inhibiting cADPR production (19, 75–79) is inconsistent with the enzymatic topology and substrate preference of CD38.

Accordingly, extracellular cyclase inhibition has limited immunoregulatory impact because: (i) CD38 performs non-sequential catalysis, yielding negligible cADPR, which is independent of ADO formation; (ii) intracellular cADPR, produced by cytosolic CD38, is inaccessible to IgG1 mAbs; and (iii) DARA- and ISA-induced enhancement of cADPR-hydrolase activity favors ADPR accumulation. Although both antibodies inhibit ADPR-Cyclase and increase cADPR-Hydrolase activity *in vitro* (**Figure 3**), extracellular CD38 continues to degrade NAD^+^ at high rates, making substantial NAD^+^ restoration unlikely (80). Moreover, the antibody-induced enzymatic shift promotes ADPR accumulation (**Figure 3D**), which can fuel the CD203a/CD73 pathway for ADO production, as shown in (27). Consequently, CD38-targeting mAbs may inadvertently sustain adenosinergic signaling *in vivo*.

*In vitro* findings support a dynamic model in which DARA enhances ADPR formation, thereby feeding into the ADO-generating pathway as previously described (27). A key limitation of this and prior studies (19, 81) is that *in vitro* assays do not fully recapitulate CD38 dynamics within the BM niche (18, 44, 52, 58, 82). Notably, *in vivo* ADPR formation—likely reflecting NAD^+^ consumption within the acidic BM microenvironment—is also detected in a BM aspirate from an MM patient who presented with very high ADO levels at diagnosis and was classified as ISS stage 3 (45) (**Supplementary Figure S2**).

In this landscape, DARA engagement induces membrane remodeling and release of MVs enriched in CD38 and co-clustered adenosinergic ectoenzymes, detectable in both BM and PB (6). These MVs provide a physiologically relevant snapshot of the enzymatic machinery at baseline and at DARA relapse (**Supplementary Figure S3**), reflecting the functional organization of the adenosinergic axis in MM. They may also contribute to the loss of surface CD38 observed in DARA-treated patients (18, 57, 71) (**Supplementary Figure S1**) and help explain inter-patient variability in adenosinergic activity at baseline and at DARA failure (**Figure 4C**). Moreover, the presence of CD26/ADA in MVs supports INO formation within the BM niche. If these cellular and vesicular mechanisms persist despite DARA-induced CD38 downregulation, they may continue to sustain the ADPR-to-ADO axis and promote conditions that favor immune escape.

In our MM cohort, the BM adenosinergic profile reflects a persistently immunosuppressive purinergic microenvironment. Mechanistically, ADO accumulation during MM progression (45) likely results from accelerated generation of NAD^+^-derived AMP and reduced ADA efficiency at low substrate concentrations. INO elevation appears context-dependent and influenced by CD26/ADA expression levels. Although ADA activity was not directly measured in BM, the detection of CD26 on patient-derived MVs supports a role for CD26-anchored ADA in this metabolic shift, as reported in melanoma and neuroblastoma (43, 48). Thus, we interpret the enhanced ADO-to-INO conversion as a biologically plausible mechanism aligned with ADA function, rather than a definitive conclusion from our dataset.

In MM, these mechanisms may be modulated by therapeutic interventions, which influence compartmental differences, nucleoside-specific trends, and inter-patient variability. To evaluate these changes *in vivo*, ADO and INO levels were measured in plasma samples obtained from BM and PB compartments at baseline and at the time of DARA treatment failure (**Figures 4A–C**). Nucleoside-specific trends at PRE-START revealed the highest concentrations, with ADO predominating in BM, confirming its role as a metabolically active niche, and INO in PB, consistent with a baseline immunosuppressive state.

During DARA treatment, BM samples showed elevated and variable ADO levels (>25 µM), concentrations sufficient to activate A2A and A2B P1 receptors and promote immunosuppression (45). However, at the time of DARA treatment failure, this ADO pool declined while remaining above immunoregulatory thresholds, whereas INO continued to accumulate (**Table 2**). In PB, nucleoside levels remained lower overall: ADO declined slightly and INO remained stable, likely reflecting systemic dilution or increased clearance. These shifts occurred without altering BM-PB metabolic ratios, indicating preserved enzymatic balance despite systemic therapy.

A marked inter-patient variability in purinergic metabolites emerged as a consistent feature of the cohort. Baseline ADO (29.66–38.70 µM) and INO (18.64–53.10 µM) levels showed wide individual ranges, reflecting the heterogeneous biological landscape of MM. This variability likely stems from differences in tumor burden, microenvironmental composition, and adenosinergic enzyme activity. Notably, outliers detected both at PRE-START and at the time of DARA treatment failure pointed to biologically distinct, potentially genetically driven MM subgroups with divergent therapeutic responses (79). Although higher BM ADO concentrations were previously associated with ISS stage 3 (45), this relationship did not readily extend to clinical PD. This is consistent with ISS capturing systemic tumor burden, whereas ADO production is shaped by niche-specific microenvironmental factors that vary substantially between patients. Indeed, representative HPLC profiles (**Figure 5**) confirmed consistent detection of ADO and INO but revealed divergent dynamics, highlighting the need for personalized metabolic monitoring.

**Figure 6:**
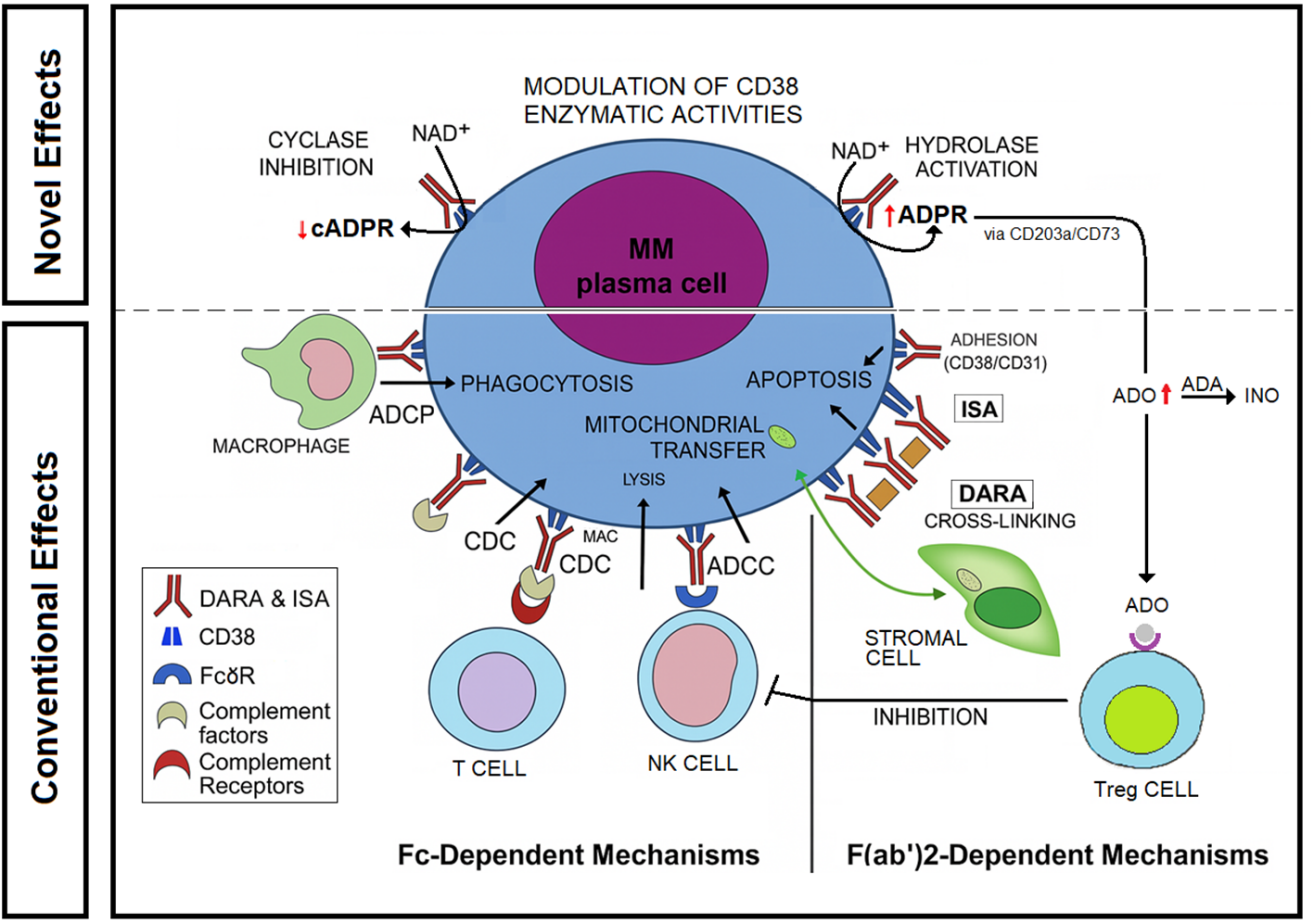
CD38 MAb-mediated effects in the BM niche. DARA and ISA target CD38 on MM cells, mediating Fc-dependent cytotoxicity and F(ab’)_2_-driven effects such as depletion of immunosuppressive cells, expansion of cytotoxic T/NK cells, inhibition of mitochondrial transfer, and induction of apoptosis (72). In addition, CD38 mAbs modulate ectoenzyme activities by reducing ADPR-Cyclase and enhancing cADPR-Hydrolase function, thereby shifting NAD^+^ metabolism toward ADPR and ADO production (27, 45). Elevated ADO promotes Treg-mediated immunosuppression (50) and is further metabolized to INO by ADA, potentially influencing therapeutic efficacy.

Current models do not fully explain the mechanisms underlying resistance to therapy (51, 83–85). DARA induces rapid and sustained depletion of CD38^+^ MM cells within hours to days after the first infusion (17), and continued dosing maintains low CD38 levels for weeks to months. At PD, CD38 expression remains reduced, contributing to diminished DARA efficacy over time (72). According to our data, this resistance may persist despite CD38 downregulation, likely due to sustained production of micromolar concentrations of immunosuppressive ADO detected in patient samples at progression and following DARA failure. Persistently high ADO levels also contribute to immune dysfunction by expanding immunosuppressive populations (Tregs, Bregs, MDSCs) and inhibiting cytotoxic T and NK cells (16). Therefore, the dual role of CD38 as both a therapeutic target and a metabolic regulator (**Figure 6**), underscore the need to further investigation into the CD38/ADA/purinergic axis to optimize anti-CD38 strategies in MM.

ADO concentrations in the BM niche are regulated not only by the CD38/CD203a/CD73 pathway (**Figure 1**) but also by clearance mechanisms (53). Among these, ADA activity in primary MM cells supports its role in ADO degradation (56, 86, 87): ADA, anchored to CD26 on MM cells and infiltrating lymphocytes (83), converts excess ADO into INO. Reduced ADA/CD26 expression impairs ADO clearance and correlates with resistance, whereas ADA overexpression in advanced MM suggests context-dependent regulation (87–89). Reported CD26 upregulation during MM progression, enhances ADA recruitment, promoting ADO-to-INO conversion and, unexpectedly, restoring effector cell cytotoxicity (90, 91). INO derived from gut microbiota further supports T-cell differentiation and improves responses to immune checkpoint inhibitors (92, 93), highlighting its dual immunoregulatory role (**Figure 1**).

INO binds A2A receptors with lower affinity than ADO and preferentially activates ERK1/2 over cAMP signaling (42, 94, 95), modulating immune responses (96). Its greater stability (half-life ~15 h vs. <10 s for ADO) (64, 87) suggests that, while ADO mediates rapid, transient effects, INO may sustain prolonged immunomodulation. Notably, ADA expression in solid tumors correlates with enhanced adaptive immunity (97), and while ADO suppresses T-cell proliferation, the ADA/CD26 complex facilitates recovery of T-cell activation (98). Thus, the immunological role of INO remains complex due to its dual regulatory functions.

Targeting the CD38/NAD^+^ axis remains an attractive immunotherapeutic strategy in MM, but the broad expression of CD38 raises concerns about off-target toxicity (99, 100), particularly in immunocompromised patients where elevated ADO may exacerbate immune dysfunction (59, 101). Among approaches to counteract ADO-mediated immunosuppression (**Supplementary Table 1**), ADA-based degradation (97, 102) is currently the most feasible, given the complexity of receptor-specific blockade (39, 50, 103), the limited potency of small-molecule CD38 inhibitors (104), and the presence of alternative ADO-generating pathways (40, 105). Emerging strategies, including biparatopic CD38 antibodies (106), NAD^+^ surrogates (73), and combinations of DARA with adenosinergic inhibitors (19, 84) or ADA-enhancing agents such as PEG-ADA (102, 107), may help mitigate ADO-driven immune escape. INO-centered interventions may also offer a safer therapeutic window, particularly in patients with low INO-producing microbiota and high ADO-associated immune suppression (92, 108).

Overall, our findings indicate that anti-CD38 therapies engage a dynamic, compartmentalized adenosinergic network in MM, with metabolic adaptations that may influence therapeutic responses. Defining how these pathways evolve under sustained CD38 pressure and identifying when adenosinergic modulation becomes clinically relevant will be essential. Integrating purinergic biomarkers into longitudinal monitoring may ultimately support more metabolically informed CD38-based immunotherapies. These considerations frame the key implications outlined in the Conclusions.

## 5 Conclusions

1) This retrospective study provides the first *in vivo* evidence that DARA monotherapy, beyond its cytotoxic effects, modulates the secondary enzymatic functions of CD38 in MM, altering ADO and INO production and supporting a model of antibody-driven purinergic remodeling. An early rise in CD38-mediated ADO production, driven by enhanced ADPR formation and independent of cADPR, appears to evolve as CD38 expression and activity adapt under therapeutic pressure. At progression, the metabolic profile indicates chronic BM niche remodeling: sustained CD38 blockade only partially limits ADO generation above immunoregulatory thresholds, while increased ADA/CD26 activity accelerates ADO deamination and shifts purine flux toward INO.

2) Larger longitudinal studies are needed to determine whether selectively targeting CD38’s enzymatic activity to limit ADO production can enhance immune responses and improve outcomes in MM, and whether the resulting ADO/INO imbalance represents a metabolic signature of resistance. Future work should also assess the value of purinergic biomarkers for monitoring therapeutic response. This study has limitations: the small number of BM samples may not capture the full heterogeneity of the disease. These considerations underscore the need for larger patient cohorts to better define the mechanisms underlying adenosinergic remodeling in MM.

## Data Availability

The original contributions presented in the study are included in the article/**Supplementary Material**. Further inquiries can be directed to the corresponding authors.
