# Supplementary material for: Modulating CD38 enzymatic activity during antibody-based immunotherapy in multiple myeloma: a basic science perspective
Source: Front Immunol. 2026 Apr 29;17:1769281. doi: 10.3389/fimmu.2026.1769281 (PMC13168026; doi:10.3389/fimmu.2026.1769281)
Supplement: Supplementary file 1 [file DataSheet1.docx]

**SUPPORTING INFORMATION**

**Modulating CD38 Enzymatic Activity During Antibody-Based Immunotherapy in Multiple Myeloma: A Basic Science Perspective**

Alberto L. Horenstein^1,2*^  Kristine A. Frerichs^3,4^ Angelo C. Faini^5^ Niels W. C. J. van de Donk^3,4^  Fabio Malavasi^1,2^

^1^Laboratory of Immunogenetics, Department of Medical Sciences, Turin, Italy; ^2^CeRMS, University of Torino, Turin, Italy and Fondazione Ricerca Molinette, Turin, Italy;

^3^Amsterdam UMC location, Vrije Universiteit Amsterdam, Department of Hematology, Amsterdam, The Netherlands;

^4^Cancer Center Amsterdam, Cancer Biology and Immunology, Amsterdam, The Netherlands;

^5^Immunogenetics and Transplant Biology Service, University Hospital "Città della Salute e della Scienza di Torino", Turin, Italy.

*Affiliation of ALH at the time of study.

**
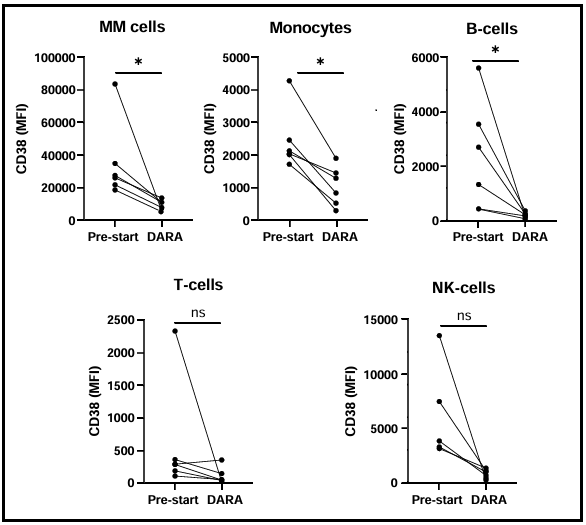
**

**Supplementary Figure S1. CD38 expression of MM cells derived from BM aspirates and immune cells subsets in PB samples from MM patients at Pre-start and during treatment with DARA monotherapy.** CD38 expression levels on monocytes, B cells, T cells, and NK cells, in sequential PB samples obtained before start of DARA monotherapy (Pre-start; n = 7), and at the time of DARA failure (DARA; n = 7). Dots represent individual expression levels. Median fluorescence intensity (MFI) is provided for each timepoint. Differences between indicated groups were calculated using Wilcoxon matched-pairs rank test. *p< 0.05; ns, not significant. Expression of CD38 was assessed using directly conjugated mAbs and analyzed on a Gallios flow cytometer (Beckman Coulter).

**
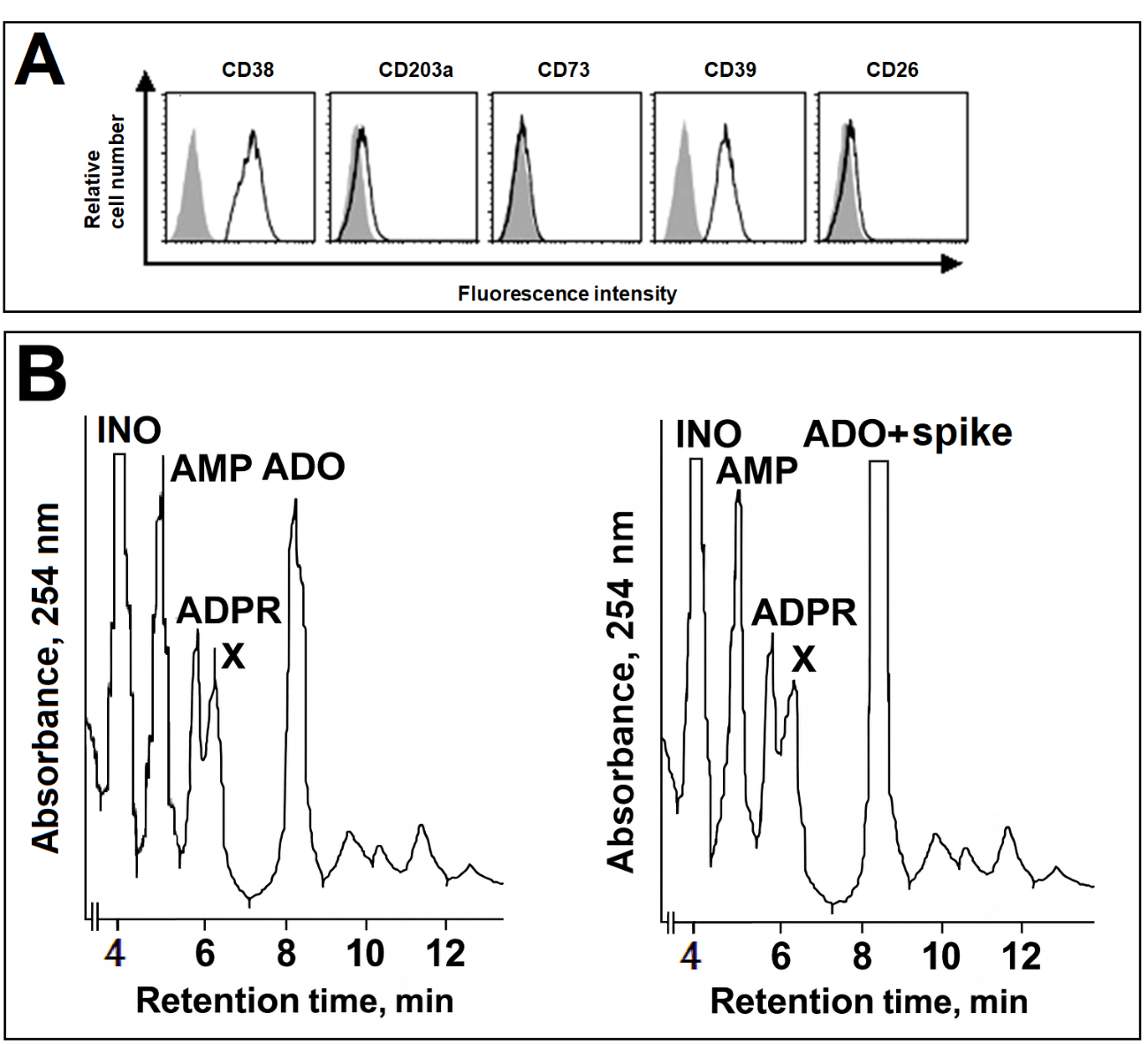
**

**Supplementary Figure S2. Purinergic activity in BM plasma from a MM patient.**

**A.** Expression of CD38, CD203a, CD39, CD73 and CD26 on primary MM cells isolated from BM aspirates using anti-CD138 mAb-coated magnetic beads. Black peaks represent marker expression; gray peaks represent isotype controls. **B**. HPLC analysis of BM plasma to assess the presence of NAD⁺ catabolites. *Left*: HPLC profile from an MM patient with very high ADO levels (X.I. = 210.84 μM) measured at diagnosis and classified as ISS stage 3. *Right*: Confirmation of ADO identity by standard spiking (50 μmol/L) in the same BM aspirate. Metabolite profiling performed using the analytical workflow applied in the present study confirms the presence of ADO, INO, and intermediate metabolites (ADPR, AMP). Peak identities were verified by co‑migration with reference standards. X: denotes an undetermined peak, likely corresponding to hypoxanthine based on retention time.

**
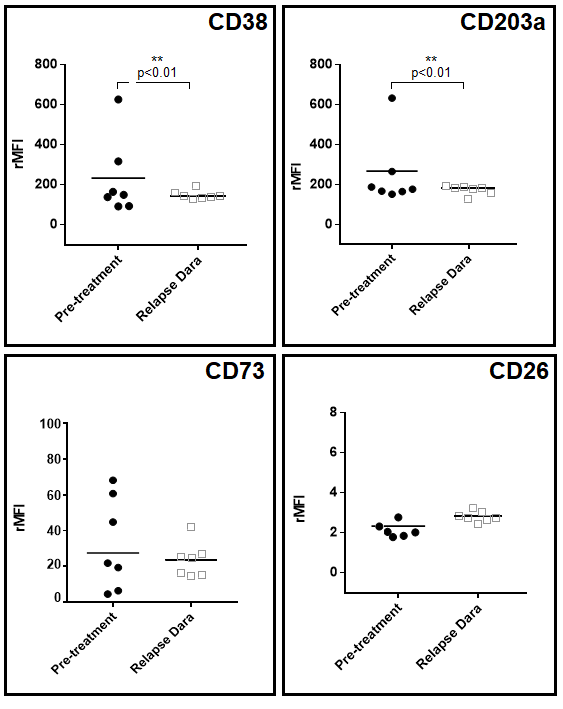
Supplementary Figure S3. Phenotypic analysis of microvesicles derived from MM cells in BM before (pre‑treatment) and after exposure to DARA (Relapse DARA), isolated from MM patients (n = 7).** Expression of selected ectoenzymes (CD38, CD39, CD203a/PC‑1, CD73, and CD26) was assessed using directly conjugated mAbs analyzed on a Gallios flow cytometer (Beckman Coulter). Black circles indicate expression levels before treatment; grey squares indicate expression levels after DARA exposure. (**)= significant at p < 0.01.

**SUPPLEMENTARY TABLE**

**Supplementary table S1.** Strategies to counteract ADO-mediated immunosuppression in MM patients treated with DARA or ISA.

**
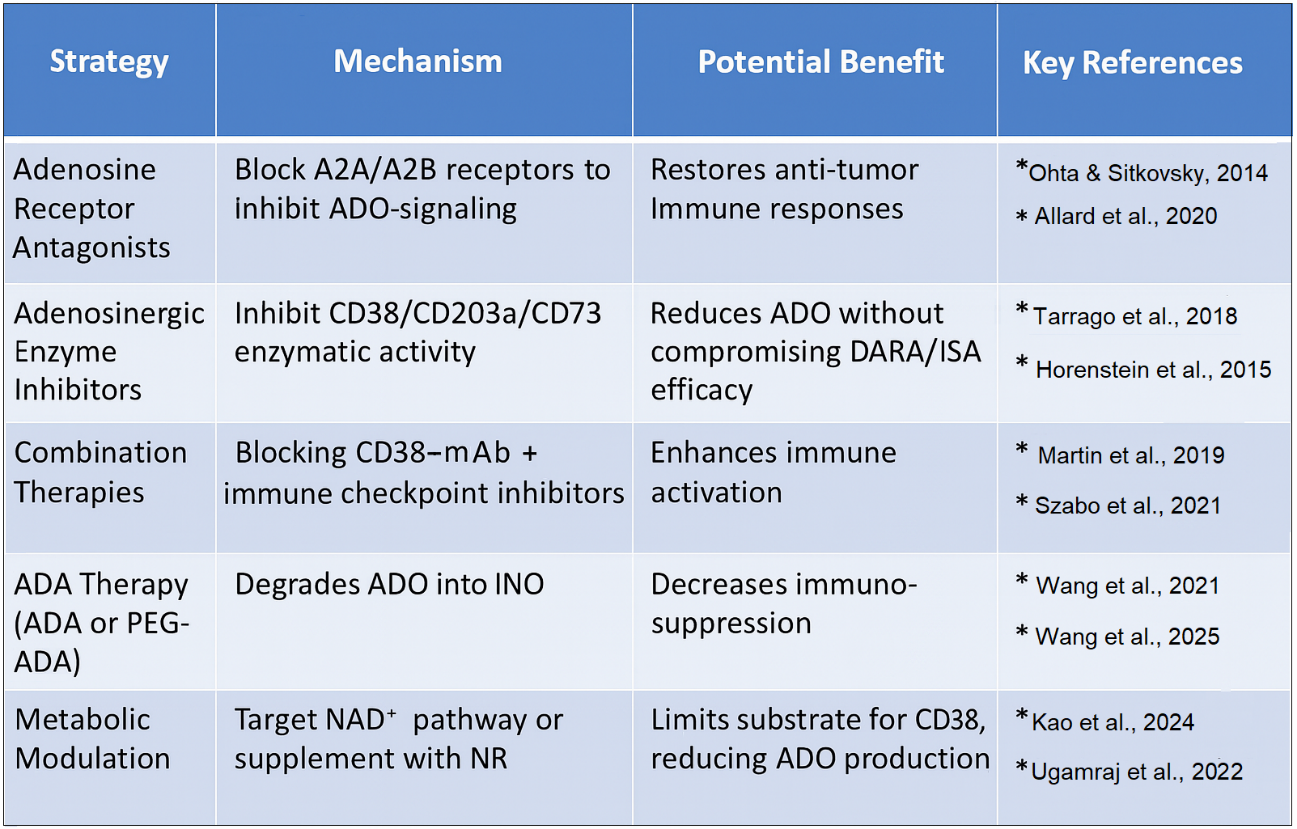
**
